# Transcriptional regulation of the *waaAE-coaD* operon by PhoP and RcsAB in *Yersinia pestis* biovar *Microtus*

**DOI:** 10.1007/s13238-014-0110-8

**Published:** 2014-10-31

**Authors:** Lei Liu, Nan Fang, Yicheng Sun, Huiying Yang, Yiquan Zhang, Yanping Han, Dongsheng Zhou, Ruifu Yang

**Affiliations:** 1 State Key Laboratory of Pathogen and Biosecurity, Beijing Institute of Microbiology and Epidemiology, Beijing, 100071 China; 2 Institute of Pathogen Biology, Chinese Academy of Medical Sciences and Peking Union Medical College, Beijing, 100730 China

**Dear Editor,**

*Yersinia pestis*, the causative agent of plague, is transmitted among mammals (including humans) primarily via the bite of infected fleas. Flea-borne transmission distinguishes *Y. pestis* from its progenitor *Y. pseudotuberculosis*, which is a mild food-borne pathogen (Zhou and Yang, 2011). In *Y. pestis*, transmission by fleas involves the synthesis of biofilms that physically block the flea’s proventriculus; *Y. pseudotuberculosis* does not produce biofilms in fleas (Zhou and Yang, 2011). Thus, biofilm formation may play a key role in virulence differences between the species.

Regulator PhoP and sensor PhoQ constitute a two-component regulatory system (Groisman, 2001). Upon environmental stimuli (such as low magnesium concentration), PhoQ transfers a phosphate group to PhoP, allowing phosphorylated PhoP (PhoP-P) to act as a transcriptional regulator. Biofilm formation is regulated by the phosphorelay system, Rcs, which is composed of three proteins: RcsB, RcsC, and RcsD. RcsC acts as the sensor kinase that catalyzes the transfer of a phosphate group to RcsD and then RcsB. Phosphorylated RcsB (RcsB-P) acts as a transcriptional regulator either independently or upon binding with an auxiliary protein RcsA, which is present in *Y. pseudotuberculosis* but not *Y. pestis.* RcsAB tightly represses biofilm formation, while the lack of RcsA in the latter results in robust biofilm formation (Sun et al., 2008).

Differences in the composition of the lipopolysaccharide (LPS) coating of the two species may also play a role in differing virulence. Due to multiple mutations in the O-antigen gene cluster, *Y. pestis* fails to produce O-antigen, a structural component of lipopolysaccharide of Gram-negative bacteria (Prior et al., 2001). An important step of LPS biosynthesis is the 3-deoxy-D-manno-octulosonic acid (Kdo) glycosylation of lipid A, which is catalyzed by the Kdo transferase WaaA (Tan and Darby, 2005). Deletion of *waaA* in *Y. pestis* leads to the reduced bacterial growth rates, a lack of Kdo in LPS, and a biofilm defect (Tan and Darby, 2005, 2006). Further study of WaaA may elucidate additional differences between the two species and their virulence.

In the present work, the RT-PCR assay indicated that the three consecutive genes *waaA*, *waaE*, and *coaD* were transcribed as a single primary RNA (Fig. S1), and thereby these three genes constituted a three-gene operon in *Y. pestis*. The relative mRNA levels of *waaA* were measured using primer extension in the wild-type *Y. pestisMicrotus* strain 201(WT) grown at 26°C or 37°C (Fig. S2A). This assay detected a single transcriptional start site (nucleotide T) located 26 bp upstream of *waaA*, and thus a single promoter was identified for *waaA* under the growth conditions tested. Cells grown at 26°C had dramatically higher levels of *waaA* mRNA than cells grown at 37°C. These results were recapitulated using a *waaA*::*lacZ* fusion vector, containing the promoter for *waaA* fused to the coding region of *lacZ*. This upregulation between 37°C and 26°C could correlate with an upregulation in response to movement from the warm-blooded host (37°C) to the flea gut (26°C). Thus WaaA may be important in the transition between the two vectors. For all the following experiments, *Y. pesti*s was cultivated at 26°C. Based on computational analysis, PhoP and RcsAB are predicted to bind the promoter proximal region of *waaAE*-*coaD*, suggesting that they may be transcriptional regulators of the operon. Primer extension experiments (Fig. 1A) indicate that a Δ*phoP* mutant has significantly lower *waaA* mRNA levels compared to WT at 26°C. A *waaA*::*lacZ* fusion strain (Fig. 1B) showed that *waaA* promoter activity was significantly reduced in Δ*phoP* relative to WT. The electrophoretic mobility shift assay (EMSA) (Fig. 1C) denoted that the purified His-PhoP protein was able to bind to the *waaA* promoter-proximal DNA in a dose-dependent manner; in contrast, His-PhoP did not bind the 16S rRNA gene at any concentration. Subsequent DNase I footprinting experiments (Fig. 1D) disclosed that His-PhoP protected a single region, located from 176 bp to 130 bp upstream of *waaA* in a dose-dependent manner. This region contained a predicted PhoP box-like sequence. Thus, PhoP positively controls *waaA* transcription through binding to the *waaA* promoter-proximal region.

**Figure 1:**
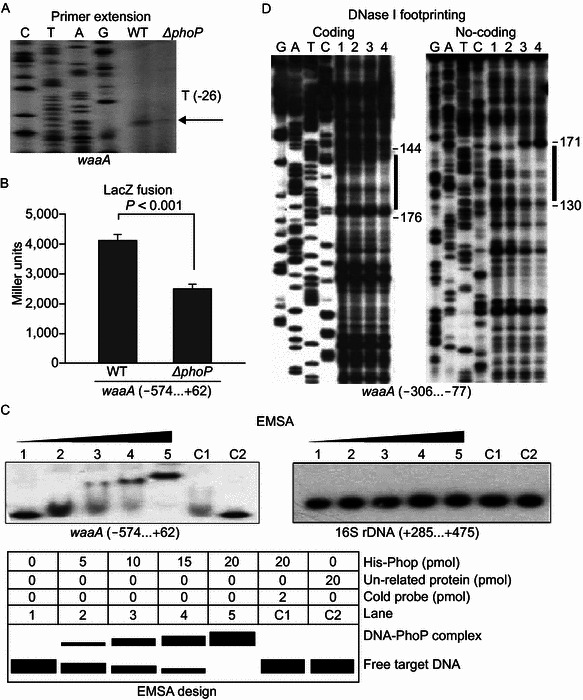
**Positive regulation of*waaAE-coaD*by PhoP**. The positive and minus numbers in the brackets indicated the nucleotide positions upstream and downstream of *waaA*, respectively. (A) Primer extension. Lanes C, T, A, and G represented Sanger sequencing reactions. The primer extension products and the sequence ladders were analyzed with an 8 mol/L urea-6% acrylamide sequencing gel. The transcriptional start site of *waaA* was indicated by arrows with nucleotides. (B) LacZ fusion. The *waaA*:*lacZ* transcriptional fusion vector was transformed into indicated *Y. pestis* strains, and then the *waaA* promoter activities (the miller units of β-galactosidase activity) were determined in the cellular extracts. (C) EMSA. The radioactively labeled DNA fragments were incubated with increasing amounts of purified His-PhoP protein and then subjected to a native 4% polyacrylamide gel electrophoresis. (D) DNase I footprinting. Labeled coding or non-coding DNA probes were incubated with increasing amounts of purified His-PhoP and then subjected to DNase I footprinting assay. The footprint regions were indicated with vertical bars. Lanes G, A, T, and C represented Sanger sequencing reactions

We created three strains to analyze the effect of RscA on binding of RscB to the *waaA* promoter: 1) *rcsA-c* containing endogenous RcsB and plasmid-borne RscA, 2) Δ*rcsB* lacking both RcsA and RscB, and 3) *rcsA-c*/Δ*rcsB* containing only plasmid-borne RscA. Compared to WT, rcsA*-c* had considerably lower levels of *waaA* mRNA, while Δ*rcsB* and *rcsA-c/*Δ*rcsB* had considerably higher levels of *waaA* mRNA (Fig. 2A). The *waaA*::*lacZ* fusion vector was introduced into the above strains, and the measurement of *waaA* promoter activity further confirmed the above primer extension results (Fig. 2B). To determine whether RcsA affects binding affinity of RcsB to the *waaA* promoter-proximal region, EMSAs were performed (Fig. 2C). His-RcsB-P alone or mixed with excess MBP-RcsA could bind to the *waaA* promoter-proximal region in a dose-dependent manner. Full DNA retardation occurred at 15 pmol with His-RcsB-P alone, whereas it was observed at 6 pmol with His-RcsB-P in presence of MBP-RcsA. These confirmed that the presence of RcsA could improve the DNA-binding activity of RcsB-P. In order to determine the location of the RcsAB binding site, DNase I footprinting experiments were performed with both coding and non-coding strands of the *waaA* promoter-proximal DNA fragment (Fig. 2D). The results showed that His-RcsB-P in the presence of MBP-RcsA protected a single region located from 41 to 18 bp upstream of *waaA.* This region contained a predicted RcsAB box-like sequence. Thus, RcsB represses *waaA* transcription by binding the promoter-proximal of *waaA* in conjunction with RcsA.

**Figure 2:**
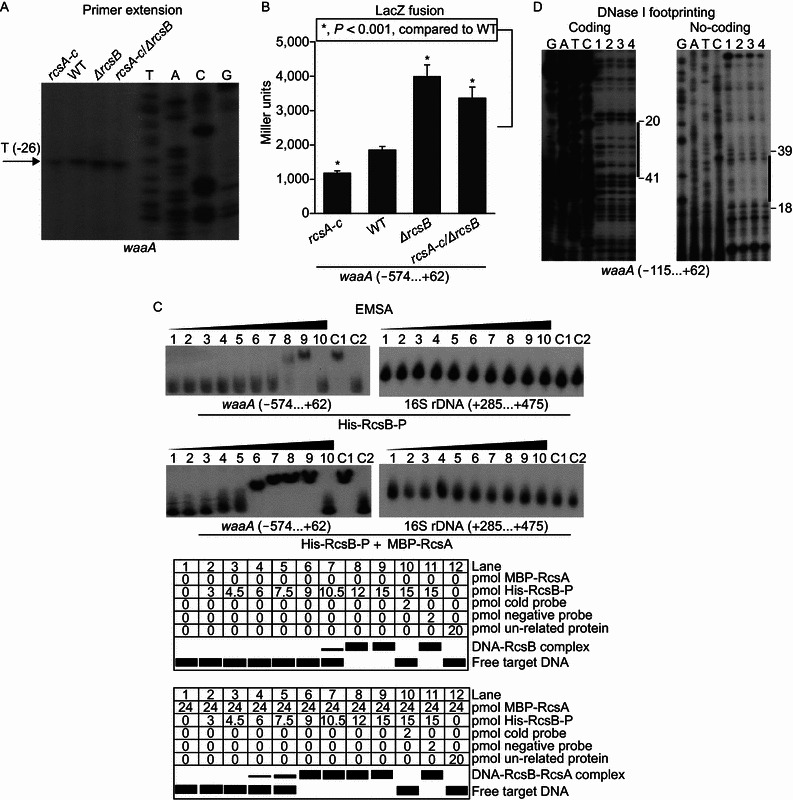
**Negative regulation of*waaAE-coaD*by RcsAB**. See Fig. 1 for the annotations of primer extension (A), LacZ fusion (B), EMSA (C), and DNase I footprinting (D) experiments. The DNA binding of His-RcsB-P in presence of MBP-RcsA (involved in EMSA and DNase I footprinting) or that of His-RcsB-P alone (EMSA) to indicated target DNA probes was tested

The structural organization of the PhoP- and RcsAB-dependent promoter of *waaA* was reconstructed based on the collected data of translation/transcription starts, predicted core promoter −10 and −35 elements, predicted Shine-Dalgarno (SD) sequences for ribosomal binding, PhoP and RcsAB sites, and PhoP and RcsAB box-like sequences (Fig. S3).

The *in vitro* biofilm masses produced by WT could be steadily stained with crystal violet (CV) staining. Compared to WT and the complement strain *C-waaA* (that showed similar levels of CV staining), Δ*waaA* stained considerably. As expected, almost no CV staining was detected for the biofilm-negative reference strain Δ*hmsS* (Fig. S4A).

Strains were incubated with nematode eggs. In the WT strain, there was abundant attachment of *Y. pestis* biofilms on nematode heads, allowing only a small portion (about 25%) of larvae to develop into L4/adult nematodes. In contrast, the bacterial lawns of Δ*waaA* and Δ*hmsS* allowed growth of 90% and 100% of nematodes, respectively (Fig. S4B). These confirmed that deletion of *waaA* led to a huge biofilm defect in *Y. pestis* biovar *Microtus*, which was consistent with the previous findings observed in biovar *Medievalis* (Tan and Darby, 2006).

When grown on LB agar, WT and *C-waaA* presented a wrinkled colony morphology due to abundant biofilm exopolysaccharide. Both Δ*waaA* and Δ*hmsS* produced very smooth colonies (Fig. S4C). This distinction indicated that deletion of *waaA* resulted in a major decrease in exopolysaccharide production. This would account for the biofilm-defective phenotype of Δ*waaA*.

The arabinose 5-phosphate (A5P) isomerase YrbH, which catalyzes the conversion of ribulose 5-phosphate into A5P, the first committed step in the Kdo biosynthesis, is required for *Y. pestis* biofilm formation (Tan and Darby, 2006). Although both of the *waaA* and *yrbH* single-gene mutants produce a truncated LPS lacking Kdo, the biofilm defects of these two strains are not identical: no trace of biofilm can be detected for the *yrbH* mutant, but small biofilms are consistently observed for the *waaA* mutant (Tan and Darby, 2006). The phosphoheptose isomerase GmhA, which is responsible for the biosynthesis of the conserved heptose component of LPS oligosaccharide core, is also required for *Y. pestis* biofilm formation and flea blockage (Darby et al., 2005). The exopolysaccharide synthesized in *Y. pestis* cells must be exported through the outer membrane, of which the predominant component is LPS. It is hypothesized that the alteration to produce a truncated LPS lacking Kdo or heptose would be most likely a cause of the dysfunction of biofilm exopolysaccharide transportation pathway in *Y. pestis*.

In addition to regulation of *waaA*, RcsAB is also known to bind to the *hmsT* promoter-proximal region to repress the transcription of *hmsT*, a gene encoding the diguanylate cyclase that is responsible for the biosynthesis of 3′,5′-cyclic diguanosine monophosphate (c-di-GMP, a second messenger promoting the production of biofilm matrix exopolysaccharide) (Sun et al., 2012). Thus, RcsAB acts as a master repressor of *Yersinia* biofilm production through inhibiting the expression of multiple biofilm determinants including at least HmsT and WaaA.

Expression of PhoP/PhoQ is induced in flea gut, where it promotes the formation of flea-borne infectious *Y. pestis* biofilms (Rebeil et al., 2013). Nevertheless, PhoP/PhoQ has no regulatory effect on the expression of *hmsHFRS*, an operon responsible for synthesis and translocation of biofilm matrix exopolysaccharides through the cell envelope (Bobrov et al., 2008). Additionally, there is no regulatory effect on *hmsHFRS*-depedent pigmentation. Moreover, PhoPQ-dependent lipid A modification, which is known to promote antimicrobial peptide resistance, plays no role in contribution of PhoP/PhoQ to *Y. pestis* biofilm formation in fleas (Rebeil et al., 2013). Data presented here indicates that *waaA* is a major determinant of *Y. pestis* biofilm production, and that *waaAE-coaD* is positively regulated by PhoP in a direct manner. Thus, PhoP is an important determinant of biofilm production in *Yersinia* and may play a role in the difference between the species *Y. pestis* and *Y. pseudotuberculosis*.

## Electronic supplementary material

Below is the link to the electronic supplementary material.

Supplementary material 1 (PDF 454 kb)
