# Supplementary material for: Transcriptional regulation of the waaAE-coaD operon by PhoP and RcsAB in Yersinia pestis biovar Microtus
Source: Protein Cell. 2014 Oct 31;5(12):940–4. doi: 10.1007/s13238-014-0110-8 (PMC4259886; doi:10.1007/s13238-014-0110-8)
Supplement: Supplementary file 1 — Supplementary material 1 (PDF 454 kb) [file 13238_2014_110_MOESM1_ESM.pdf]

Materials and Methods

Bacterial strains. The wild-type *Yersinia pestis Microtus* strain 201 (WT) is avirulent to humans but highly virulent to mice [1]. The entire or partial coding region of each indicated gene was replaced by the kanamycin resistance cassette by using the one-step inactivation method based on the lambda phage recombination system [2], to generate the corresponding null mutants of *Y. pestis* (Table S1). For *in trans* gene complementation, a PCR-generated DNA fragment containing the coding region of *Y. pestis waaA* or functional *Y. pseudotuberculosis rcsA* together with its promoter-proximal region and transcriptional terminator-proximal region was cloned into the low-copy-number cloning vector pBBR1MCS-5 [3] or pACYC184 (GenBank accession number X06403), respectively. The resulting recombinant vector pBBR1MCS-5-*waaA* or pACYC184-*rcsA* was transformed into each indicated *Y. pestis* strain lack of functional *waaA* or *rcsA* for *in trans* complementation of *waaA* or *rcsA*, respectively (Table S1). All the primers designed in this study were listed in Table S2.

Bacterial growth and RNA isolation. Overnight cell culture in the Luria-Bertani (LB) broth with an optical density (OD_{620}) of about 1.0 was diluted 1:50 into 18 ml of the fresh LB broth for further cultivation at 26°C with shaking at 230 rpm to reach an OD_{620} of about 1.0. In order to elicit temperature upshift, half of the cell cultures were incubated at 37°C with shaking at 230 rpm for 3 h, and the remaining half were allowed to grow continuously at 26°C for 3 h. These two kinds of cultures were simply designated as bacteria grown at 37 and 26°C, respectively. Immediately before bacterial harvest, double-volume of RNAprotect reagent (Qiagen) was added to one-volume of cell culture. Total RNA was extracted using TRIzol Reagent (Invitrogen).

Primer extension assay. As described previously [4, 5], a 5'-^{32}P-labeled oligonucleotide primer complementary to a portion of the *waaA* RNA transcript was employed to synthesize cDNAs from total RNA templatesusing a Primer Extension System (Promega). If different *Y. pestis* strains were involved in a single experiment, the same amount of total RNA was used as starting materials. Sequence ladders were prepared with the same 5'-^{32}P-labeled primers using AccuPower & Top DNA Sequencing Kit (Bioneer). Radioactive species were detected by autoradiography. The 5'-terminus of *waaA* RNA transcript (i.e., the *waaA* transcription start) was mapped according to the size of primer extension product, while the relative *waaA* mRNA levels

were determined with the intensities of primer extension product.

LacZ fusion and β-galactosidase assay. A promoter-proximal DNA region of *waaA* was cloned into the low-copy-number transcriptional fusion vector pRW50 [6] that harbors a promoterless *lacZ* reporter gene. As described previously [4, 5], *Y. pestis* strains transformed with the recombinant plasmid or the empty pRW50 (the negative control) were grown as described above to measure the β-galactosidase activity in the cellular extracts using the β-Galactosidase Enzyme Assay System (Promega).

Protein expression and purification. The entire coding regions of *Y. pestis phoP*, *Y. pseudotuberculosis rcsA*, and *Y. pestis rcsB* were cloned into plasmids pET28a (Novagen) [7], pMAL-c4X (Invitrogen) [8], pBADMyc-His A (New England Biolabs) [8], respectively. *E. coli* BL21λDE3 was used as the host cells for overexpression of 6×His-tagged PhoP (His-PhoP) [7], while the wild-type *Y. pestis* strain KIM6+ and the *rcsB* null mutant of KIM6+ were employed for maltose-binding protein (MBP)-tagged RcsA (MBP-RcsA) and His-RcsB, respectively [8]. His-PhoP [7] or His-RcsB [8] was purified under native conditions using a Ni-NTA Agarose Column (Qiagen), while MBP-RcsA [8] using a Amylose Agarose Column (New England Biolabs). Each of the purified proteins was dialyzed and then concentrated to a concentration of about 0.1 mg/ml in phosphate buffered saline (pH 8.0) containing 20% glycerin.

Electrophoretic mobility shift assay (EMSA). Each indicated 5'-^{32}P-labeled target DNA fragment was incubated with increasing amounts of purified His-PhoP or His-RcsB, or with increasing amounts of purified His-RcsB with addition of 24 pmol of purified MBP-RcsA, for 30 min at room temperature in a binding buffer as described previously [4, 5]. To achieve RcsB phosphorylation, 25 mM fresh acetyl phosphate was added in the binding buffer and incubated with purified His-RcsB for 30 min before adding labeled DNA probes. The resulting reactions were subjected to a native 4% (w/v) polyacrylamide gel electrophoresis. Each EMSA experiment included three controls, namely, cold probe as the specific DNA competitor (the same promoter-proximal DNA region unlabeled), negative probe as the nonspecific DNA competitor (the unlabeled coding region of the 16S rRNA gene), and nonspecific protein competitor (rabbit anti-F1-protein polyclonal antibodies) [4, 5]. Sequencing and radioactive species detection were as above.

DNase I footprinting. For DNase I footprinting [4, 5], the target DNA fragment with a single ^{32}P-labeled end was incubated with increasing amounts of purified His-PhoP alone or His-RcsB-P with addition of 24 pmol of purified MBP-RcsA, which

was followed by partial digestion of RQ1 RNase-Free DNase I (Promega). The digested DNA samples were purified and analyzed in an 8 M urea-6% polyacrylamide gel. Sequencing and radioactive species detection were as above. Footprints were identified by comparison with the sequence ladders.

Biofilm assays. Three different methods [9] were used to detect *Y. pestis* biofilms: i) crystal violet staining of the *in vitro* biofilm masses that were attached to the well walls when bacteria were grown in polystyrene microtiter plates; ii) determination of the percentages of fourth-stage larvae and adults (L4/adult) of *C. elegans* after incubation of nematode eggs on *Y. pestis* lawns, which negatively reflected the bacterial ability to produce biofilms; and iii) observation of the rugose colony morphology of bacteria grown on LB agar plates, which positively reflected the bacterial ability to synthesize biofilm matrix exopolysaccharide.

Prediction of *waaAE-coaD* as direct PhoP and RcsAB targets. Known or predicted RcsAB box-like sequences in *Enterobacteriaceae* were collected (Table S3) and aligned to generate two RcsAB consensus constructs (Fig. S5): an updated RcsAB box sequence TAAGAAT-ATTCTTA (a 7-7 invert repeat), and a position frequency matrix (PFM) recording the position-dependent frequency of each nucleotide. A PFM described a sequence motif more precisely a box (string) sequence, because a PFM was modeled from a set of box-like sequences.

The PFM representing conserved DNA signals recognized by PhoP [10] or RcsAB (see above) was used for consensus matching within the 300bp upstream region of *waaA* [11]. This analysis predicted a PhoP box-like sequence 5'-TATTTATTGATTTTTTAT-3' (located from 151 to 168 bp upstream of *waaA*) with a weight score of 9.04, and also a RcsAB box-like sequence 5'-TAAGAATAATACCA-3' (from 28 to 41 bp upstream) with a score of 7.88. Higher score value indicated higher probability of regulator-target promoter recognition, and the detecting score values were larger than the frequently used cutoff value of seven. The above computational promoter analysis suggested that PhoP and RcsAB could recognize the promoter-proximal region of *waaAE-coaD*.

Experimental replicates and statistical methods. For LacZ fusion, crystal violet staining of biofilms, and determination of L4/adult nematodes, experiments were performed with at least three independent bacterial cultures/lawns, and the values were expressed as mean ± standard deviation. Paired Student's *t*-test was performed to determine statistically significant differences; $P < 0.01$ was considered to indicate statistical significance. For primer extension, EMSA, and DNase I footprinting, and

colony morphology observation, representative data from at least two independent biological replicates were shown.

Table S1 *Y. pestis* strains involved in gene deletion and complementation

Strain	Functional (+) or inactivated (-)					Feature	Reference
	rcsA	***rcsB***	***waaA***	***phoP***	***hmsS***		
WT	-	+	+	+	+	The wild-type *Y. pestis* biovar *Microtus* strain 201. The *rscA* gene was inactivated naturally.	[1]
c-rcsA	+	+	+	+	+	The vector pACYC184-*rcsA* was introduced into WT.	This study
ΔrcsB	-	-	+	+	+	The entire coding region of *rcsB* gene was deleted from WT.	This study
ΔrcsB/c-rcsA	+	-	+	+	+	The vector pACYC184-*rcsA* was introduced into *ΔrcsB*.	This study
ΔphoP	-	+	+	-	+	The base pairs 41 to 631 of *phoP* gene was deleted from WT.	[4]
ΔwaaA	-	+	-	+	+	The entire coding region of *waaA* gene was deleted from WT.	This study
c-waaA	-	+	+	+	+	The vector pBBR1MCS-5-*waaA* was introduced into *waaA*.	This study
ΔhmsS	-	+	+	+	-	A reference biofilm-negative strain. The base pairs 146 to 468 of *hmsS* was deleted from WT.	[12]

Table S2 Oligonucleotide primers designed in this study

Target	Primers (forward/reverse, 5′-3′)
**Gene mutation**	
rcsB	AACTTGATGCCAACGTGCTAATTACTGACCTCTCTATGCCAGATTGCAGCATTACACG/GTTACCAGAAAACCTTCAGCAAAGAGTCGCAAAACTTCACTGTAACGCACTGAGAAGC
waaA	ATGAATAACCACGGCTCAATGTGGTGCTCCGCCCTCCTAGGTGTAGGCTGGAGCTGCTTC/TTAGTGGCTCCGTTGTGGCAGATAAGGCTCCAACAAATGTCATATGAATATCCTCCTTA
**Gene complementation**	
rcsA	GCGGTCGACCGGCAGCACAGCGTAAAC/ GCGGGATCCGGTCACTACTCAGGCAAGAAAG
rcsB	GCGGTCGACGAGCGGCGAATTTTATCTGAAC/ GCGGGATCCGTTTGCGGCAGTTGAATCAC
waaA	CGCGGATCCGTGCAAAAGCCGTAGTC/CCCAAGCTTCGATTTTAAGCTCTGGG
**RT-PCR**	
rfaC-waaA intergenic	GAACATTTCCCACACGTAGAGG/ AGTAAACGCAGCCAAATCAGAG
waaA-waaE intergenic	TGTTGGCGGTAGTTTGGTTG/ GGCATATTGTTGAGCGAGTTG
waaE-coaD intergenic	GCCATTCTCAGCCATACTCTTG/ GAACTGTCCGCAATAGCCAAG
**LacZ fusion**	
waaA	GCGGGATCCTGGATCGCCCAAACATTACG/GCGAAGCTTAAACGCAGCCAAATCAGAGG
**Primer extension**	
waaA	GTAAACGCAGCCAAATCAGAG
**EMSA**	
waaA	TGGATCGCCCAAACATTACG/AAACGCAGCCAAATCAGAGG
**DNase I footprinting**	
waaA for PhoP binding	GCACCCAACAGCTCTAACG/GCGGAGCACCACATTGAG
waaA for RcsAB binding	TCTTCCTATGAATAACCACG/GCGAAGCTTAAACGCAGCCAAATCAGAGG

Table S3. Collection of RcsAB box-like sequences

Bacterium	Gene	RcsAB box-like sequence	Reference
**Exopolysaccharide synthesis and regulation**			
Escherichia coli/Shigella	*wza*	TAAAGAAACTCCTA	[13, 14]
	yjbE	TGAGGTTAATCCTA	[15]
	galF	TAAGATTATTCTCA	[16]
Salmonella enterica	*tviA*	TAGGAATATTCTTA	[13]
Klebsiella pneumoniae	*galF*	TAAGGAAATTCTGA	[13, 17]
Y. pseudotuberculosis /Y.pestis	*hmsH*	TAAGAATAATCCTA	[12]
	hmsT	TAAGAAAAATCCTA	[12]
	YPO0450	TAAGATAAATCTCA	[12]
Erwinia amylovora	*amsG*	TGAGAATAATCTTA	[13, 18]
Pantoea stewartii	*cpsA*	TGGAATAAATCTGA	[13, 18]
**Mobility**			
E. coli	*flhD*	TAGGAAAAATCTTA	[19]
S. enterica		TAGGAAAAATCTTA	Predicted
Y. enterocolitica		TAGGAATAATCCTA	Predicted
Y. pseudotuberculosis		TAGGAATATTCCTA	Predicted
**RcsA**			
E. coli/Shigella	*rcsA*	TAAGGATTATCCGA	[13, 20]
S. enterica		TAAGGTTTATCCGA	[13, 21]
K. pneumoniae		TAAGGAAATTCTGA	[13, 22]
Y. pseudotuberculosis		TACGGATTTTCCGA	Predicted
E. amylovora		TAAGAATAGTCCTA	[13, 23]

Shigella species, *K. pneumoniae*, and *Y. pestis* are negative for motility due to the absence of *flhDC* or the presence of nonfunctional *flhDC*. *rcsA* is inactivated in *Y. pestis*.

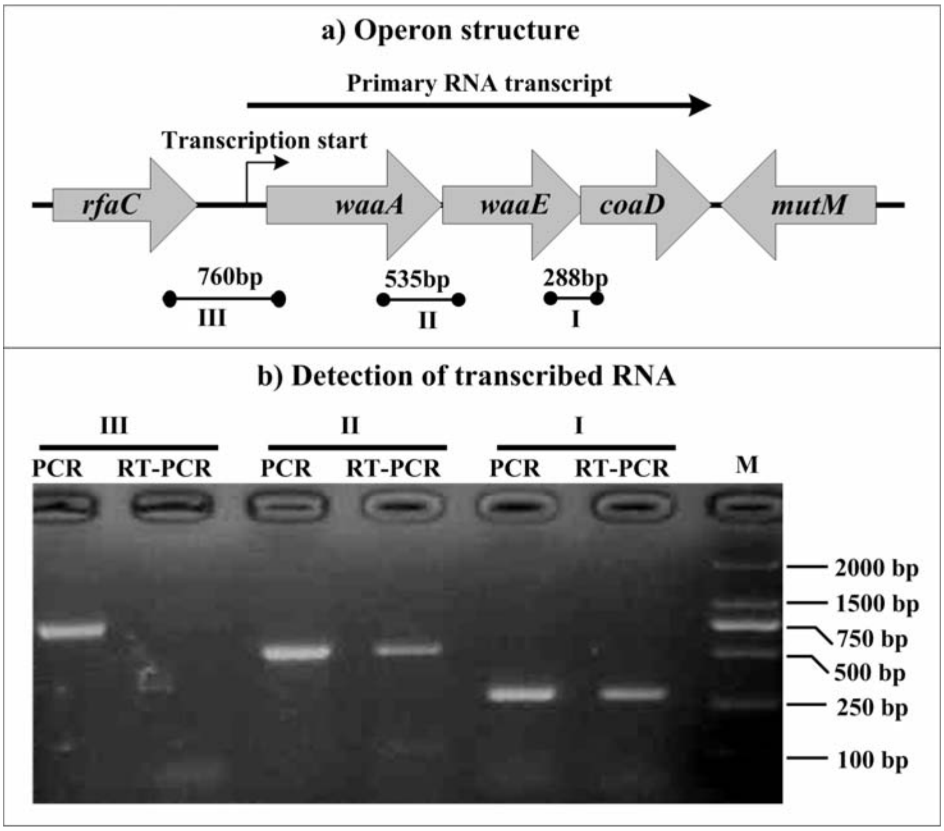

Fig. S1 Transcriptional organization of *waaA*, *waaE* and *coaD*. a) Operon structure. The boxed arrows represented the length and direction of indicated ORFs. The broken arrows indicated the transcriptional start (i.e., transcribed promoters). The arrowheads indicated the location of primer pairs and the expected PCR amplicons. The horizontal arrows depicted the putative primary RNA transcript transcribed for the *waaAE-coaD* operon . **b) Detection of transcribed RNA.** The cDNA samples were generated by reverse transcription (RT) from total RNA of WT grown at 26°C. Genomic DNA and cDNA were used as the templates for PCR and RT-PCR, respectively. To ensure that no contamination of genomic DNA in the RT reactions would occur, RT-PCR of negative controls was performed using the “cDNA” sample generated without reverse transcriptase as template. Reactions containing primer pairs without templates were also included as blank controls. As expected, both negative and blank controls of RT-PCR produced no amplicon (data not shown).

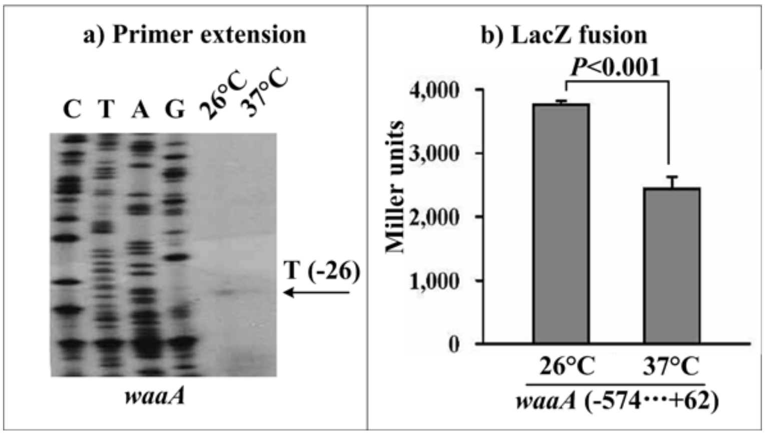

Fig. S2 Down-regulation *of waaA* upon temperature upshift from 26 to 37°C. The minus and positive numbers in the brackets indicated nucleotide positions upstream and downstream of *waaA*. **a) Primer extension.** Lanes C, T, A, and G represented Sanger sequencing reactions. The primer extension product for WT grown at 26 or 37°C and the sequence ladders were analyzed with a 8 M urea-6% acrylamide sequencing gel. The transcriptional start site of *waaA* was indicated by arrows with nucleotides. **b) LacZ fusion.** The *waaA*:*lacZ* transcriptional fusion vector was transformed into WT, and then the *waaA* promoter activities (the miller units of β-galactosidase activity) were determined in the cellular extracts of WT grown under 26 or 37°C.

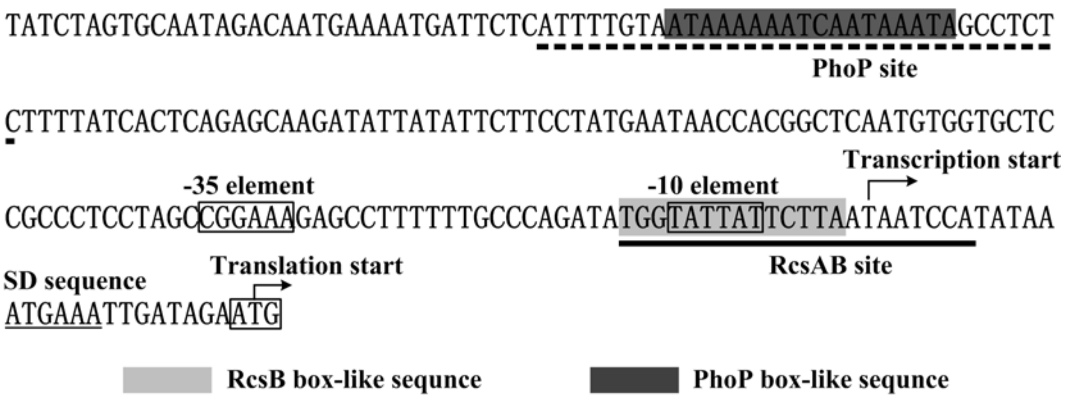

Fig. S3 Structure of promoter-proximal DNA region of *waaA*. Shown were translation/transcription starts, predicted core promoter -10 and -35 elements, predicted Shine-Dalgarno (SD) sequences for ribosomal binding, PhoP and RcsAB sites, and PhoP and RcsAB box-like sequences.

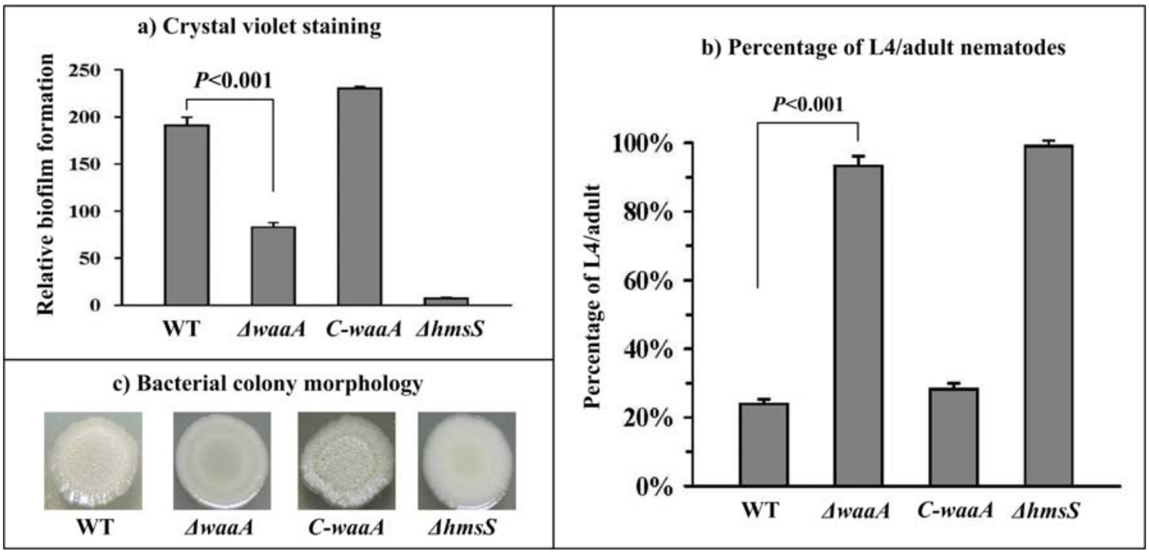

Fig. S4 *Y. pestis* biofilms assays. a) Crystal violet staining of *in vitro* biofilms. *Y. pestis* was grown in the 24-well polystyrene dishes, and the bacterial biomass adherent to the well walls were stained with crystal violet to determine the OD_{570} values. The planktonic cells were subjective to determine the OD_{620} values. The relative capacity of biofilm formation of each strain tested was shown with the OD_{570}/OD_{620} values. **b) Bacterial colony morphology.** Aliquots of bacterial glycerol stocks were spotted on the LB plate, followed by the incubation for one week. **c) *Yersinia* biofilms on nematodes.** After incubation of nematode eggs on the lawns of indicated *Y. pestis* strains, the developmental stages of nematodes on each lawn were scored to calculate the percentage of L4/adult.

(a) Sequence-Logo

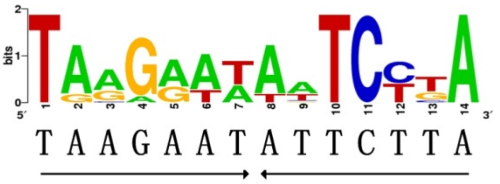

(b) PFM

A	0	16	12	2	12	14	8	16	11	0	0	0	0	19
C	0	0	1	0	0	0	0	0	1	0	19	10	2	0
G	0	3	6	17	7	0	0	0	1	0	0	0	6	0
T	19	0	0	0	0	5	11	3	6	19	0	9	11	0

Fig. S5 RcsAB consensus constructs. (a) The sequence logo of aligned RcsAB box-like sequences (Table S3). (b) The PFM denoting the frequency of each nucleotide (raw) at each nucleotide position (column).

References

1. Zhou D, Tong Z, Song Y, Han Y, Pei D, Pang X, Zhai J, Li M, Cui B, Qi Z *et al*: **Genetics of metabolic variations between Yersinia pestis biovars and the proposal of a new biovar, microtus**. *J Bacteriol* 2004, **186**(15):5147-5152.
2. Datsenko KA, Wanner BL: **One-step inactivation of chromosomal genes in Escherichia coli K-12 using PCR products**. *Proc Natl Acad Sci U S A* 2000, **97**(12):6640-6645.
3. Kovach ME, Elzer PH, Hill DS, Robertson GT, Farris MA, Roop RM, 2nd, Peterson KM: **Four new derivatives of the broad-host-range cloning vector pBBR1MCS, carrying different antibiotic-resistance cassettes**. *Gene* 1995, **166**(1):175-176.
4. Zhang Y, Wang L, Fang N, Qu S, Tan Y, Guo Z, Qiu J, Zhou D, Yang R: **Reciprocal regulation of pH 6 antigen gene loci by PhoP and RovA in Yersinia pestis biovar Microtus**. *Future Microbiol* 2013, **8**(2):271-280.
5. Zhang Y, Wang L, Han Y, Yan Y, Tan Y, Zhou L, Cui Y, Du Z, Wang X, Bi Y *et al*: **Autoregulation of PhoP/PhoQ and Positive Regulation of the Cyclic AMP Receptor Protein-Cyclic AMP Complex by PhoP in Yersinia pestis**. *J Bacteriol* 2013, **195**(5):1022-1030.
6. Lodge J, Fear J, Busby S, Gunasekaran P, Kamini NR: **Broad host range plasmids carrying the Escherichia coli lactose and galactose operons**. *FEMS Microbiol Lett* 1992, **74**(2-3):271-276.
7. Zhang Y, Gao H, Wang L, Xiao X, Tan Y, Guo Z, Zhou D, Yang R: **Molecular characterization of transcriptional regulation of rovA by PhoP and RovA in Yersinia pestis**. *PLoS One* 2011, **6**(9):e25484.
8. Sun YC, Guo XP, Hinnebusch BJ, Darby C: **The Yersinia pestis Rcs phosphorelay inhibits biofilm formation by repressing transcription of the diguanylate cyclase gene hmsT**. *J Bacteriol* 2012, **194**(8):2020-2026.
9. Fang N, Gao H, Wang L, Qu S, Zhang YQ, Yang RF, Zhou DS: **Optimized methods for biofilm analysis in Yersinia pestis**. *Biomed Environ Sci* 2013, **26**(5):408-411.
10. Li YL, Gao H, Qin L, Li B, Han YP, Guo ZB, Song YJ, Zhai JH, Du ZM, Wang XY *et al*: **Identification and characterization of PhoP regulon members in Yersinia pestis biovar Microtus**. *BMC Genomics* 2008, **9**(1):143.
11. van Helden J: **Regulatory sequence analysis tools**. *Nucleic Acids Res* 2003, **31**(13):3593-3596.
12. Sun F, Gao H, Zhang Y, Wang L, Fang N, Tan Y, Guo Z, Xia P, Zhou D, Yang R: **Fur is a repressor of biofilm formation in Yersinia pestis**. *PLoS One* 2012, **7**(12):e52392.
13. Wehland M, Bernhard F: **The RcsAB box. Characterization of a new operator essential for the regulation of exopolysaccharide biosynthesis in enteric bacteria**. *J Biol Chem* 2000, **275**(10):7013-7020.
14. Stout V: **Identification of the promoter region for the colanic acid polysaccharide biosynthetic genes in Escherichia coli K-12**. *J Bacteriol* 1996, **178**(14):4273-4280.
15. Ferrieres L, Aslam SN, Cooper RM, Clarke DJ: **The yjbEFGH locus in Escherichia coli K-12 is an operon encoding proteins involved in exopolysaccharide production**. *Microbiology* 2007, **153**(Pt 4):1070-1080.
16. Rahn A, Whitfield C: **Transcriptional organization and regulation of the Escherichia coli K30 group 1 capsule biosynthesis (cps) gene cluster**. *Mol Microbiol* 2003, **47**(4):1045-1060.

17. Carlier AL, von Bodman SB: **The rcsA promoter of Pantoea stewartii subsp. stewartii features a low-level constitutive promoter and an EsaR quorum-sensing-regulated promoter**. *J Bacteriol* 2006, **188**(12):4581-4584.

18. Wehland M, Kiecker C, Coplin DL, Kelm O, Saenger W, Bernhard F: **Identification of an RcsA/RcsB recognition motif in the promoters of exopolysaccharide biosynthetic operons from Erwinia amylovora and Pantoea stewartii subspecies stewartii**. *J Biol Chem* 1999, **274**(6):3300-3307.

19. Francez-Charlot A, Laugel B, Van Gemert A, Dubarry N, Wiorowski F, Castanie-Cornet MP, Gutierrez C, Cam K: **RcsCDB His-Asp phosphorelay system negatively regulates the flhDC operon in Escherichia coli**. *Mol Microbiol* 2003, **49**(3):823-832.

20. Ebel W, Trempy JE: **Escherichia coli RcsA, a positive activator of colanic acid capsular polysaccharide synthesis, functions To activate its own expression**. *J Bacteriol* 1999, **181**(2):577-584.

21. Virlogeux I, Waxin H, Ecobichon C, Lee JO, Popoff MY: **Characterization of the rcsA and rcsB genes from Salmonella typhi: rcsB through tviA is involved in regulation of Vi antigen synthesis**. *J Bacteriol* 1996, **178**(6):1691-1698.

22. Stout V, Torres-Cabassa A, Maurizi MR, Gutnick D, Gottesman S: **RcsA, an unstable positive regulator of capsular polysaccharide synthesis**. *J Bacteriol* 1991, **173**(5):1738-1747.

23. Bernhard F, Poetter K, Geider K, Coplin DL: **The rcsA gene from Erwinia amylovora: identification, nucleotide sequence, and regulation of exopolysaccharide biosynthesis**. *Mol Plant Microbe Interact* 1990, **3**(6):429-437.
